# La dépression post-accident vasculaire cérébral au Burkina Faso

**Published:** 2012-09-03

**Authors:** Christian Napon, Arnaud Kaboré, Jean Kaboré

**Affiliations:** 1 Service de neurologie, CHU Yalgado Ouedraogo, BP 7022, Ouagadougou 03, Burkina Faso

**Keywords:** Dépression, accident vasculaire cérébrale, Afrique, Burkina Faso, Depression, Stroke, Africa, Burkina Faso

## Abstract

**Introduction:**

Lourde de conséquences sur la récupération motrice du patient, la dépression post-accident vasculaire cérébral (DPAVC) est souvent méconnue et sous-diagnostiquée. Notre objectif était d'en étudier les aspects épidémiologiques, cliniques, thérapeutiques en milieu hospitalier au Burkina Faso.

**Méthodes:**

Il s'agissait d'une étude transversale de 21 mois. Elle a concerné tous les cas d'AVC hospitalisés durant la période d’étude dont le délai de survenue de l'ictus était supérieur ou égal à 2 semaines. Une fois le diagnostic de DPAVC posé par les critères du DSM IV, sa sévérité était évaluée par l’échelle MADRS (Montgomery and Asberg Depression Rating Scale). Les patients DPAVC étaient revus 2 mois pour réévaluer leur dépression.

**Résultats:**

Sur 167 hospitalisations pour AVC, 65 patients avaient une DPAVC (38,9%). L’âge moyen était de 56,9 ans avec des extrêmes de 29 et 84 ans. Le sex ratio était de 0,9. Les AVC ischémiques étaient majoritaires (53,8%). Quarante-trois patients (66,15%) présentaient une DPAVC mineure et 22 patients (33,85%), une DPAVC majeure. La DPAVC a été précoce dans 73,8% des cas (délai inférieur ou égal à 30 jours). Un traitement antidépresseur a concerné 28 patients (43,1%). L’évolution à deux mois chez 31 patients était favorable dans 29% des cas.

**Conclusion:**

La DPAVC est fréquente dans notre contexte. Elle compromet le pronostic fonctionnel et augmente le risque de morbidité et de mortalité Elle devrait être recherchée systématiquement chez tout hémiplégique, en particulier à la phase précoce, lors des entretiens réguliers avec la famille, et devant toute modification du psychisme du patient.

## Introduction

La dépression post-accident vasculaire cérébrale (DPAVC) n'a été considérée comme une entité nosologique que depuis les années 1980 [1]. Manifestation psychiatrique la plus fréquente après un accident vasculaire cérébral (AVC), elle se définit comme une dépression survenant dans un contexte d'AVC. Lourde de conséquences tant au niveau de ses implications sociales, sur la qualité de vie, mais aussi sur les possibilités de récupérations motrices du patient, sa prévalence estimée est de 30 à 35% avec des extrêmes allant de 20 à 60% [2, 3]. Méconnue et sous diagnostiquée, la DPAVC n'avait jamais été décrite dans notre contexte. Notre objectif était d'étudier les aspects épidémiologiques, cliniques, thérapeutiques de la DPAVC dans le service de Neurologie du CHU Yalgado Ouédraogo de Ouagadougou au Burkina Faso.

## Méthodes

Il s'agissait d'une étude transversale, de 21 mois allant du 06 Février 2009 au 14 Novembre 2010, effectuée dans le service de neurologie du CHU Yalgado Ouédraogo. Cette étude a concerné tous les patients victimes d'AVC, confirmés par la tomodensitométrie cérébrale, dont le délai depuis la survenue de l'ictus était supérieur ou égal à 2 semaines. Au préalable un consentement éclairé était obtenu après un entretien avec le patient. Une fois le diagnostic de DPAVC posé à partir des critères du DSM IV, sa sévérité était évaluée par l'échelle MADRS (Montgomery and Asberg Depression Rating Scale). La dépression était mineure si le score MADRS était entre 16 et 30 points, et majeur si le score MADRS était supérieur à 30. Les patients DPAVC étaient revus 2 mois après l'ictus pour réévaluer leur dépression. Les données ont été recueillies, sur des fiches de collecte établies à cet effet, à partir d'informations recueillies auprès des malades et de leurs accompagnants. Les variables étudiées étaient: l'âge, le sexe, le type d'AVC, le siège anatomique de la lésion, les signes neurologiques, la sévérité de la DPAVC, son délai d'apparition et le traitement reçu, La saisie et l'analyse des données ont été effectuées à l'aide du logiciel Epi-info version 3.4.3. L'étude a été approuvé par le département de Médecine et spécialités de l'UFR (Unité de Formation et de Recherche) Sciences de la Santé et le comité d'éthique du Centre Hospitalier Universitaire Yalgado Ouédraogo.

## Résultats

Sur un effectif de 167 patients hospitalisés pour AVC au cours de la période d'étude, 65 patients ont présenté une DPAVC soit 38,9% des patients. L'âge moyen était de 56,9 ans avec des extrêmes de 29 et 84 ans. Le sexe féminin a représenté 52,3% des cas de DPAVC dans notre étude, soit un sex-ratio de 0,9. Le niveau d'instruction était majoritairement bas. Sur les 65 cas de DPAVC, 30 patients étaient analphabètes, 14 patients avaient un niveau d'instruction primaire, 20 étaient de niveau secondaire et 1 patient de niveau supérieur. A la TDM cérébrale, la lésion vasculaire a intéressé l'hémisphère cérébral gauche chez 38 patients (58,5%), et l'hémisphère droit chez 27 patients (41,5%). Les AVC ischémiques étaient les plus nombreux (53,8%), suivis des hémorragies cérébrales (44,6%), la thrombose veineuse cérébrale représentait 1,5% des cas. Pour les AVC ischémiques (n=35), le siège anatomique le plus fréquemment rencontré était le territoire de l'artère cérébrale moyenne chez 28/35, suivies de la cérébrale antérieure (3/35), la cérébrale postérieure (2/35), la choroïdienne antérieure (2/35). Le handicap moteur était présent chez 63/65. Il s'agissait d'une hémiplégie chez 43 patients (66,7%) et d'une hémiparésie chez 20 patients (30,2%). Le Tableau 1 montre la répartition des principaux signes cliniques neurologiques chez les patients DPAVC en fonction de leur fréquence. Selon le score MADRS, 43/65 patients DPAVC avaient une dépression mineure soit un score situé entre 16 et 30 et 22/65 une dépression majeure avec un score>30. Le délai d'apparition était précoce (inférieur ou égal à 30 jours) chez 48/65 patients et tardif (>30 jours) chez les 17/65 restants. Sur le plan thérapeutique, 28/65 patients ont bénéficié d'un traitement antidépresseur. Le Tableau 2 montre la répartition des DPAVC selon le traitement reçu. L'évolution à 2 mois après l'hospitalisation a été appréciée chez 31/65 patients, les 34/65 autres n'ayant pas respecté leur consultation de suivi. 22/65 patients présentaient toujours une DPAVC et 9/35 n'étaient plus déprimés.

**Tableau 1:** Répartition des principaux signes cliniques des DPAVC en fonction de leur fréquence

Signes neurologiques	Effectif	En%
Déficit moteur	63	96
Troubles sensitifs	15	23
Troubles du langage	11	17
Dysarthrie	5	7

**Tableau 2:** Répartition des DPAVC en fonction du traitement administré

Traitement	Effectif	%
Paroxétine	13	46,5
Amitriptyline	2	7,2
Méprobamate	1	3,5
Psychothérapie seule	9	32,1
Psychothérapie + Amitriptyline	1	3,5
Psychothérapie + Paroxétine	2	7,2
**Total**	**28**	**100**

DPAVC : Dépression Post accident vasculaire cérébral

## Discussion

La prévalence moyenne de la DPAVC est élevée. Elle est estimée à environ 30-35%, avec des extrêmes allant de 20 à 60% [3]. Ce taux est conforme aux résultats auxquels nous sommes parvenus qui est de 38,9%. Cependant, cette grande variabilité du taux de prévalence (20 à 60%) de la DPAVC est essentiellement due, selon Paolucci et al. à la méthodologie utilisée incluant les critères diagnostiques, le type d'échelle de dépression, et le délai de l'évaluation du patient après l'AVC [3]. Notre étude a également mis en évidence une prédominance féminine (52,3%). En effet, les facteurs de risque prémorbides liés à la DPAVC sont d'abord constitutionnels touchant avec prédilection les patients de sexe féminin. Toutefois, on peut retrouver également d'autres facteurs de risque prémorbides cliniques (antécédents d'AVC et leur gravité, épisodes de troubles dépressifs ou psychiatriques, déficience cognitive ou aphasie préexistantes, personnalité névrosée, histoire familiale de dépression). Le bas niveau d'instruction comme dans notre série, l'apathie, la réaction de déni à la phase aiguë, et l'atrophie cérébrale ont été aussi rapportés comme facteurs de risque de la DPAVC [3, 4]. La prépondérance des lésions vasculaires gauches dans la survenue de la DPAVC comme l'illustre notre série, a été rapportée par d'autres auteurs [5]. Les études menées à l'aide de la tomographie par émission de positons ont mis en évidence une réduction du métabolisme des régions sous-corticales gauches [6] identifiées comme siège de prédilection de l'AVC pour la survenue de la dépression. L'hémisphère gauche serait spécialisé dans le contrôle des émotions positives et l'hémisphère droit des émotions négatives. En cas de dysfonctionnement de l'hémisphère gauche, il y'aurait prépondérance du contrôle de l'hémisphère droit avec libération des symptômes dépressifs [7]. Le handicap moteur est déterminant dans la survenue de la DPAVC (63/65 dans notre série). En effet, la perte brutale, relative (hémiparésie) ou totale (hémiplégie) de l'usage des membres, constitue un lourd tribut moral pour le patient qui se voit perdre son autonomie. Ce constat a conduit SNAPHAAN L [8] à affirmer que l'indépendance fonctionnelle a un effet protecteur contre la DPAVC. Notre étude a montré une précocité dans le délai de survenue de la DPAVC qui était inférieur à 30 jours à compter de la date de l'AVC dans 73,8% des cas. Ces résultats sont superposables à ceux d'autres auteurs [9] et trouveraient leurs explications dans le stress lié à la nouvelle vie à l'hôpital, le caractère soudain et imprévu de l'installation du déficit, le désarroi et la grande inquiétude qui entourent le patient et ses proches durant la phase aigue. Malgré ce délai précoce de survenue de la DPAVC dans la majorité des cas, notre étude a aussi montré que la DPAVC était mineure chez la plupart des patients (43/65) selon le score MADRS. Ceci résulte de la mise en place par l'entourage du malade d'un dispositif efficient de soutien psychologique (psychothérapie interpersonnelle) sous-tendu par une solidarité interpersonnelle particulièrement remarquable, permettant de canaliser et d'atténuer l'humeur dépressive du patient DPAVC.

## Conclusion

La DPAVC est fréquente mais de sévérité mineure dans notre contexte. Elle compromet le pronostic fonctionnel et augmente le risque de morbidité et de mortalité. Sa spécificité réside dans un ensemble de particularités sémiologiques: survenue précoce après l'AVC, lien avec certaines localisations encéphaliques. Cette étude appelle à notre réflexion que la DPAVC devrait être recherchée systématiquement chez tout hémiplégique post-AVC, en particulier à la phase précoce, lors des entretiens réguliers avec la famille, et devant toute modification du psychisme du patient.
